# Diseases of Women Unconnected with Pregnancy and the Puerperal State

**Published:** 1845-12

**Authors:** 


*Diseases of Women unconnected with Pregnancy and the Puerperal state.*—1. *Amenorrhœa.*—The disorders connected with the function of menstruation never fail to command a considerable share of attention. The different aspects which deficiency of this excretion may assume, are well described by Mr. Bell, in a paper published in a recent number of the Edinburgh Medical and Surgical Journal. In reference to the modus operandi of emmenagogue medicines, this author observes that much less assistance has been derived from the more correct physiological notions respecting the function of menstruation which have been established during the last few years, than might reasonably have been expected; in fact, that the theory of the periodic maturation and expulsion of ova so far from rendering the action of medicines more intelligible, has tended rather to the greater obscurity of the subject. We cannot, however, admit that the explanation of the action of these medicines as derived from their operation upon the blood, is at all less applicable under our improved physiological knowledge, than it had previously been; for it is just as easy to conceive that a due constitution of that fluid is necessary to the perfect maturation of an ovum, as to the healthy formation of the bile. We do not mean to assert that there is any close resemblance between the production of ova and the secretion of bile, but merely that as both constitute the essential function of the ovarium and the liver respectively, a healthy state of the blood is as necessary in the one case as in the other, and the action of medicines, therefore, equally intelligible.

The comparative power of the different preparations of iron in restoring the blood in chlorotic patients to its normal state, is noticed by MM. Raciborski and Selade; by the latter in an elaborate paper on the therapeutic value of the ferruginous preparations in general. M. Raciborski agrees with MM. Quevenne and Miquelard in giving the preference to metallic iron in an extremely minute state of division, the mode of obtaining which will be found in another part of this work. The memoir of M. Selade is very complete of its kind, and enters minutely into the value of each individual preparation of iron. The best of these, according to this author, are the proto-muriate or hydrochlorate, the carbonate and the lactate; into which latter it is believed by Gelis and Contè, that the other preparations are converted by the action of the gastric juice. This opinion, however, is denied by M. Selade, who leans to the assumption that the metal enters into combination with the free muriatic acid of the stomach. The question is one of too purely a chemical nature for this report, and we therefore shall pass on to something of a more practical nature. We may, however, before dismissing the subject, briefly allude to the author's theory of the mode in which the iron combines with the blood. When, according to his theory, a preparation of iron becomes absorbed, it is quickly decomposed, and its ferruginous portion combines with the globules which have lost their hæmatosin, and which in this condition are found in company with great numbers of colorless globules. If it has already been oxydized, it soon becomes converted into the carbonate of the protoxide by uniting with the carbonic acid of the venous blood. When these globules arrive at the lungs, they absorb oxygen from the atmosphere, and the iron passes at once to the state of peroxide, the carbonic acid being given off.

Although chlorosis and amenorrhœa are not necessarily connected, we cannot do better than here allude to the description by M. Ricord, of a form of chlorosis depending upon syphilitic contamination. It has been ascertained that the action of the syphilitic poison is principally exercised upon the globules, in the number of which it causes a considerable diminution, for which reason ferruginous preparations are in general in these cases of great efficacy. They are not, however, as has been observed by M. Ricord sufficient by themselves, as in simple chlorosis, but require at the same time a special anti-syphilitic medication. M. Ricord, therefore, employs mercurials as well as iron, but we may remark that no combination is more likely to be of use in these cases than a combination of the latter metal with iodine.

2. *Menorrhagia.*—In the treatment of profuse menstruation, the oxide of silver is very highly spoken of by Sir James Eyre in a useful little work lately published. According to the author's experience, it is superior to the secale cornutum, gallic acid, and indeed all other remedies. The dose is half a grain three times a day, gradually increased to one or two grains. A new, and according to M. Givestet, very active medicine in the same disease is the expressed juice of the *urtica urens*. Our own experience in the treatment of menorrhagia is greatly in favor of the ergot of rye, which we give in the dose of 10 grains of the powder with 6 grains of the pulv. ipecac. c. We have seldom met with a case unconnected with organic disease of the uterus which has not yielded speedily to this combination; and we may state, moreover, that having been in the habit of giving it for five or six years, we have never met with any symptom whatever to lead us to apprehend the least inconvenience from its employment.

We may likewise observe that the active principle of the secale called ergotin has been productive of the greatest benefit in the uterine hemorrhages depending upon malignant disease. Dr. Ebers, who has had extensive experience of the medicine, recommends the following formula when the ergotin cannot be procured. Bruised ergot 100 grammes, boiling water 500 grammes, macerate for three hours and strain; then add 5 grammes of fresh lemon juice.

3. *Vicarious menstruation.*—Three cases of this rare, though sufficiently familiar occurrence, have been lately recorded. The first is that of a female who having received a lacerated wound near the elbow, had from it a supplementary periodic discharge for several months in succession. Upon her becoming pregnant the wound partially cicatrized, but the scab fell off subsequently to her delivery, again giving issue to a periodic discharge of blood, which continued until the menstrual function became thoroughly re-established. The second instance is reported in the New York Journal of Medicine. The third was brought before the Société d'Emulation of Paris by M. Forget. The supplementary discharge in this case was from the conjunctiva, auditory passages, and from certain portions of the integuments.

4. *Diseases of the Ovaria—Acute Ovaritis.*—The occurrence of acute inflammation of the ovaries, independently of the puerperal state, is generally thought to be unfrequent, so much so that it appears to be treated of in systematic works on the Diseases of Females, rather because its omission would leave a hiatus in the nosological code than from any distinct conception in the minds of the authors of its existence as a separate disease. There is little doubt, however, that these organs are liable to attacks of inflammation in the virgin state; and it is probable that, if the diagnosis could be rendered less complicated, it would be found to be a commoner cause of many of the symptoms attendant upon dysmenorrhœa, and other sexual affections in females than is generally suspected. The subject has lately been illustrated in an elaborate memoir by M. Chereau, which has since been published in a separate form. The author acknowledges three conditions under which acute ovaritis may develop itself; first, and by far the most frequent, the puerperal state; secondly, immediately before and after the menstrual period; and thirdly, from extension of disease from neighboring parts, as the rectum, uterus,

&c. The disease may terminate in resolution, induration, softening, suppuration, and gangrene, the former being, under appropriate treatment, the most usual event. The inflamed ovary may occasionally be perceived by palpitation of the abdominal walls, but more frequently, unless greatly enlarged, it cannot be so recognised. A surer diagnosis may be formed either by vaginal examination, or by an examination per rectum, which latter is the most certain, as we are able by that means to make direct pressure upon the diseased organ. M. Chereau in the enumeration of the symptoms omits one which we have always found to be present in well-marked cases of the disease, viz: pain upon the passage of the hardened fæces. We have several times found patients laboring under dysmenorrhœa and sudden suppression of the menses, in which cases we believe that the ovaries are often inflamed, who have complained of a great aggravation of their sufferings whenever the bowels have been allowed to become costive. The treatment recommended bythe author does not need particular mention.

5. *Ovarian Tumor.*—The subject of ovarian diseases of a chronic character have lately been invested with a peculiar interest, from the attempt that has been made to revive the operation of gastrotomy for their removal. We do not regret that we have no case to record of this operation which has occurred during the period embraced by our report. The merits of the operation itself have, however, met with a considerable attention at the hands of several persons well qualified to give an opinion, among whom we may mention Mr. Liston, Dr. Ashwell, &c., and the result has been its unequivocal condemnation by the majority, as unjustifiable in any point of view in which it can be considered. The remarks of Mr. Liston may be fonnd in the Lancet, Feb. 8, 1845. Dr. Ashwell, after taking a general review of the progress of ovarian tumors, thus expresses himself: "It does not appear that statistics more favorable even than we have a right to expect, will materially change the aspect of the circumstances under which the operation is to be performed. * * * * What would be thought of the feasibility of any other operation involving life in imminent hazard, if it were discovered that out of *sixty-seven* cases, it was fro.n *absolute error in diagnosis incapable of completion in eighteen;* that of the remaining forty-nine, where the extirpation was effected, *sixteen died*, and *two were not cured;* so that out of the whole number *the operation failed in thirty-six, and succeeded in thirty-one*, less than one half."

The editor of the London and Edinburgh Monthly Journal does not give a more promising view of the operation than the writer above quoted; indeed, as given by the former, the statistics of the operation place it possibly still lower in the scale of feasibility. In 89 cases collected in a tabular form in the above named Journal, it appears that 55 recovered and 34 perished from the immediate consequences of the operation. But it appears also that in 9 cases either no tumor was found when the abdomen was laid open, or that it was unconnected with the ovary—cases which place the certainty of diagnosis in a very unfavorable light—and moreover in 14 others the operation could not be completed. Of these 23 patients, 13 recovered and 10 died; so that the number of instances in which the intentions of the operator were fully carried out is reduced to 65, of which number 25 died. This, it certainly appears to us, is a mortality which, although it should not deter from operating in a disease by which life must otherwise inevitably be speedily sacrificed, should make us pause before we interfere in a malady which may be endured in many cases throughout a long life without other inconvenience than that arising from the bulk of the tumor.

The last writer we shall mention by whom the operation of ovariotomy has been considered, is M. Cazeau, in a memoir on the subject presented to the Société d'Emulation of Paris, at the meeting of the 4th Dec., 1844. The opinions expressed by this author correspond in every respect with those above mentioned*

Turning from the disheartening evidence with respect to operative measures for the cure of ovarian tumors, we refer with pleasure to a case reported by Mr. Brown, in which a perfect cure followed the use of compression combined with the exhibition of mercurials and diuretics. This case, as Mr. Brown very justly observes, should encourage us to greater energy in the medical treatment of these diseases.

---

*It is but right to state, that since the above was written, the operation of ovariotomy has been strenuously supported by Mr. Atlee (Amer. Jour. of the Med. Sciences, April, 1845,) in some remarks appended to an account of a successful case of abdominal section for the extirpation of a fibrous tumor of the uterus. The statistics of the operations, as stated by him, are the most extensive of any as yet on record, amounting to 101 cases, in which 38 did, or 1 in 2⅝. The causes of death in the fatal cases are from hemorrhage in 8, peritonitis in 8, exhaustion in 2, inflammation of large intestines in 1, gangrene 2, peritonitis and gangrene 1, causes not stated 16, total 38.

A case related by Mr. Hardy in the same journal, in which a large ovarian tumor, having twice impeded delivery, ultimately caused death by inducing strangulation of the intestine, and another by Dr. Lambrecht, in which the contents of an ovarian cyst were on two separate occasions discharged through the umbilicus.

